# Herbal hot compress combined with bianstone warm ironing improves anxiety and cognitive impairment via gut-brain-HPA axis in pubertal mesenteric lymphadenitis rats

**DOI:** 10.3389/fped.2026.1929229

**Published:** 2026-09-04

**Authors:** Lei An, Zhanyong Li, Chundan Zhang, Wei Tan, Wei Sun

**Affiliations:** 1 Department of Pediatrics, The First Affiliated Hospital of Guizhou University of Traditional Chinese Medicine, Guiyang, China; 2 Department of Emergency Medicine, The First Affiliated Hospital of Guizhou University of Traditional Chinese Medicine, Guiyang, China; 3 School of Life Sciences, Langfang Normal University, Langfang, China; 4 Department of Neonatology, The First Affiliated Hospital of Guizhou University of Traditional Chinese Medicine, Guiyang, China

**Keywords:** bianstone therapy, cAMP, cognitive function, gut-brain axis, mesenteric lymphadenitis

## Abstract

**Introduction:**

Pediatric mesenteric lymphadenitis (ML) is characterized by persistent intestinal inflammation, intestinal barrier damage and excessive oxidative stress, which can disturb the gut-brain axis, overactivate the hypothalamic-pituitary-adrenal (HPA) axis, and induce anxiety and cognitive disorders in children. Herbal hot compress (HC) and bianstone warm ironing (BS) are clinically used to relieve related symptoms, yet their mechanism linking intestinal oxidative stress and gut barrier function remains insufficiently clarified. This study aimed to investigate combined HC and BS effects on intestinal oxidative stress indicators (MDA, SOD), intestinal barrier markers (DAO, D-lactic acid, LPS), gut-brain-HPA axis function, hippocampal cAMP, neurotransmitter levels, and synaptic plasticity in pubertal ML rats, and verify the cAMP/PKA signaling pathway.

**Methods:**

Briefly, pubertal rats were randomly divided into six groups. ML was induced by repeated LPS injection (2 mg/kg) once every 3 days for 5 consecutive cycles. After 14 days of intervention, EPM, OFT, and NOR tests evaluated anxiety-like behaviors and cognitive function, while *in vivo* hippocampal LTP recordings at the Schaffer collateral-CA1 pathway assessed synaptic plasticity, including I/O curves and PPR. Serum samples were collected to detect oxidative stress, intestinal barrier parameters, inflammatory cytokines and HPA hormones; hippocampal neurotransmitters and cAMP were measured via ELISA.

**Results:**

Model rats exhibited intestinal oxidative injury, barrier dysfunction, gut inflammation, HPA hyperactivity, neurotransmitter imbalance, impaired synaptic plasticity, and obvious anxiety and cognitive deficits. No intergroup differences in I/O curves and PPR were observed across six groups. BS or HC monotherapy partially ameliorated these abnormalities, and HC exerted stronger effects on improving intestinal oxidative stress and gut barrier integrity. Combined HC + BS achieved superior outcomes to single treatments in restoring intestinal redox balance, protecting intestinal barrier and alleviating gut-brain-HPA axis dysfunction. Pretreatment with a PKA antagonist largely abolished these protective effects.

**Discussion:**

In conclusion, herbal hot compress combined with bianstone warm ironing alleviates anxiety and cognitive impairment in pubertal ML rats by inhibiting intestinal oxidative injury, repairing barrier damage, suppressing inflammation, normalizing HPA axis, restoring neurotransmitter homeostasis and improving hippocampal synaptic plasticity, partially mediated by the cAMP/PKA pathway.

## 1 Introduction

Pediatric mesenteric lymphadenitis (ML) is a common digestive system disorder in school-age children, manifesting as recurrent abdominal pain, abdominal distension, and loss of appetite (1). Accumulating evidence demonstrates that chronic intestinal inflammation induced by ML disrupts the bidirectional gut-brain axis, triggers excessive hypothalamic-pituitary-adrenal (HPA) axis activation, and further causes central nervous system dysfunction in children (2, 3). Intestinal oxidative stress imbalance, presented with elevated malondialdehyde (MDA) and decreased superoxide dismutase (SOD), aggravates intestinal mucosal oxidative injury and inflammatory cascade. Meanwhile, intestinal barrier damage increases gut permeability, leading to the release of intestinal lipopolysaccharide (LPS) into circulation, accompanied by increased serum diamine oxidase (DAO) and D-lactic acid, which are classic biomarkers of intestinal barrier dysfunction (4, 5).

Children with persistent ML frequently suffer from comorbid anxiety, inattention and sleep disturbances, severely affecting physical and mental development (1, 3, 6). Puberty is a critical period for hippocampal maturation and neurotransmitter system establishment; inflammatory stress during this stage disrupts synaptic plasticity and neurotransmitter homeostasis, resulting in long-term emotional and cognitive impairments (7–9). Herbal hot compress (HC) and bianstone warm ironing (BS) are classic external therapies of traditional Chinese medicine. Clinical and preliminary studies have verified their regulatory effects on intestinal inflammation and central emotional pathways, with reliable clinical applicability (10, 11). Single application of HC or BS can partially relieve intestinal and emotional symptoms, yet their standalone efficacy remains limited and unstable. Combined treatment exhibits better therapeutic potential and synergistic effects in clinical practice.

Nevertheless, several critical research gaps remain unclear. First, existing studies only reported the peripheral intestinal protection or behavioral improvement of single HC or BS intervention. No preclinical study has systematically compared the differential efficacy of HC monotherapy, BS monotherapy, and their combination on intestinal oxidative stress, intestinal barrier function, gut-brain-HPA axis homeostasis and hippocampal synaptic plasticity in pubertal ML models (12–14). Second, the synergistic mechanism of combined HC and BS therapy linking peripheral intestinal repair and central emotional-cognitive improvement has not been clarified. Third, although the cAMP (cyclic adenosine monophosphate)/PKA (protein kinase A) pathway is a core regulator of gut-brain axis communication and hippocampal synaptic function (15–18), its mediating role in TCM combined external therapy against ML-induced gut-derived emotional and cognitive deficits during puberty remains unvalidated.

The cAMP/PKA signaling pathway serves as a key second messenger system regulating hippocampal neurotransmitter synthesis, synaptic plasticity and gut-brain-HPA axis balance (19–21). Dopamine (DA) modulates emotional stability and attention, while the glutamate (Glu)/*γ*-aminobutyric acid (GABA) ratio reflects central excitatory-inhibitory homeostasis (22–25). Hippocampal long-term potentiation (LTP) is the gold-standard indicator for evaluating synaptic plasticity and cognitive function (26, 27).

In this study, a pubertal ML rat model was established via repeated LPS injection. Behavioral tests, ELISA and *in vivo* electrophysiology were applied to systematically detect intestinal oxidative stress, intestinal barrier function, inflammation, HPA axis activity, hippocampal neurotransmitter levels and synaptic plasticity. We further compared the efficacy of HC, BS and their combination, and applied a PKA antagonist to verify the cAMP/PKA-dependent mechanism. We hypothesized that HC combined with BS exerts synergistic effects to alleviate ML-induced anxiety and cognitive impairment via regulating intestinal injury and gut-brain-HPA axis dysfunction, partially through activating the cAMP/PKA pathway. This study fills the above research gaps, clarifies the synergistic gut-brain regulatory mechanism of combined TCM external therapy, and provides preclinical evidence for the clinical treatment of pediatric gut-derived emotional-cognitive comorbidities.

## 2 Experimental procedure

### 2.1 Experimental animals

Forty-eight SPF male pubertal Sprague Dawley rats were purchased from a qualified laboratory animal center. All rats were housed in independent ventilated cages under standardized conditions: 12 h light/dark cycle, ambient temperature 22 ± 2 ℃, relative humidity 50 ± 5%, with free access to autoclaved chow and filtered water. After 7 days of adaptive feeding, rats were randomly divided into 6 groups (*n* = 8) using a random number table: Control group, Model group, HC group, bianstone warm ironing (BS) group, HC + BS group, and Antagonist + HC + BS group. All experimental procedures were reviewed and approved by the Institutional Animal Care and Use Committee of Langfang Normal University (SCXK-2019-0010) and performed in strict accordance with the ARRIVE guidelines and national laboratory animal welfare regulations.

Upon arrival, all rats underwent an at least 5-day adaptive feeding period, during which brief daily gentle handling and restraint acclimatization in the customized soft elastic plastic pouch were performed simultaneously. This pre-experimental habituation was completed before rats reached 35 days of age, ensuring that all subsequent model establishment, interventions, and tests were conducted strictly within the pubertal stage (35–42 days old).

### 2.2 Model establishment

Rats in the Model, HC, BS, HC + BS, and Antagonist + HC + BS groups received intraperitoneal injection of LPS (2 mg/kg) once every 3 days for 5 consecutive cycles to induce persistent intestinal inflammation and mesenteric lymph node (MLN) enlargement. This intermittent low-dose LPS stimulation protocol is a well-validated classic modeling method for pubertal ML, which stably induces intestinal inflammatory infiltration, mesenteric lymph node immune activation, lymphoid tissue hyperplasia and macroscopic lymph node enlargement in rodents, and has been widely adopted and verified by macroscopic weighing, histological staining and molecular phenotypic identification in previous preclinical studies (28–32). Rats in the Control group were injected with equal volume of sterile normal saline and raised routinely. Upon completion of experiments, MLNs were carefully dissected under a stereomicroscope (Olympus Corporation, Tokyo, Japan), cleared of adherent fat and weighed to obtain MLN weight for MLN index, which was calculated as: MLN index (%) = (MLN wet weight/body weight) × 100%.

### 2.3 Drug treatment and group assignment

Twenty-four hours after the last LPS injection, all interventions were initiated and administered once daily at a fixed time for 14 consecutive days. For the HC group and BS group, each single intervention session lasted 30 min. For the HC + BS group and Antagonist + HC + BS group, rats received sequential combined intervention: 30 min of herbal hot compress immediately followed by 30 min of Bianstone warm ironing, with a total daily intervention duration of 60 min. The surface temperature of the herbal compress and bianstone in direct contact with the skin was maintained at 42–45°C and continuously monitored and maintained via a calibrated infrared thermometer (Fluke 62 MAX, Fluke Corporation, Everett, WA, USA, accuracy ±1.5°C) during the entire intervention. This temperature range is a validated safe and effective mild thermal stimulation range for pubertal rodent external thermal therapy in consistent with previous analogous preclinical studies (33, 34). During the entire intervention period, rats were gently restrained in a laboratory-customized soft elastic plastic restraint pouch with reserved openings for head and four limbs to allow limited free movement of the head and extremities and minimize handling stress. No anesthesia was administered. The pouch was sized to fit pubertal rats, with adjustable Velcro fasteners on the lateral sides to avoid excessive thoracic/abdominal compression and respiratory obstruction. Rats maintained natural posture without sustained struggling during thermal stimulation. To prevent thermal injury to rat skin and superficial tissues, multiple standardized protective measures were implemented. First, strict control of constant mild thermal stimulation at 42–45°C to avoid high-temperature scalding. Second, uniform paving of herbal paste and stable preheating of bianstone to ensure consistent and balanced surface temperature without local overheating. Third, fixed single intervention duration to eliminate tissue damage caused by prolonged thermal stimulation. Fourth, real-time observation of rat skin status after each intervention, and no thermal injury-related skin lesions were observed in any experimental group throughout the experiment.

#### 2.3.1 Control group and model group

To ensure consistent handling stress and identical experimental manipulation conditions across all six groups and eliminate behavioral and physiological confounding factors derived from differential artificial intervention, strict standardized sham procedures were performed in parallel with treatment groups throughout the 14-day intervention cycle. Rats in both control and model groups were restrained in the identical customized soft elastic plastic pouch with a unified daily handling duration of 60 min, matching the maximum manipulation duration applied to the HC + BS combined intervention group. During daily sham intervention, a blank non-heated 4 cm × 6 cm sterile gauze pad (free of herbal powder and thermal treatment) was affixed to the abdominal acupoints CV8 (Shenque), ST25 (Tianshu), and CV12 (Zhongwan). Meanwhile, non-heated bianstone was used for gentle simulated warm ironing along the Governor Vessel from GV14 (Dazhui) to GV1 (Changqiang). Sham Bianstone simulation followed the same stroking route, rhythm, pressure, and frequency as the real BS intervention, only without heating. All rats received unified non-thermal tactile sham stimulation without herbal or thermal intervention, excluding confounding influences caused by differential handling exposure.

#### 2.3.2 HC group

A herbal compress paste (Rhizoma Atractylodis Preparata 10 g, Rhizoma Atractylodis Macrocephalae Preparata 10 g, Pericarpium Citri Reticulatae 6 g, Fructus Crataegi Carbonisatus 10 g, Fructus Hordei Germinatus Preparatus 10 g, Endothelium Corneum Gigeriae Galli Preparatum 5 g, Spica Prunellae 10 g, Semen Coicis Crudum 12 g, Rhizoma Dioscoreae Preparata 15 g, Rhizoma Curcumae Vinegar-processed 5 g, Fructus Toosendan Preparatus 10 g) was prepared as follows: each crude herb was pulverized into fine powder and mixed thoroughly in the specified ratio. The mixed powder (total 5 g per rat) was blended with warm water (1:1, w/v) to form a paste, then spread evenly onto a 4 cm × 6 cm gauze pad to create a rat-sized compress pouch. The compress pouch was secured to the abdominal acupoints CV8 (Shenque), ST25 (Tianshu), and CV12 (Zhongwan) using a self-adhesive non-woven medical bandage, which was wrapped loosely around the abdomen to prevent displacement while avoiding excessive compression of the thorax and abdomen. The pouch was applied to the acupoints CV8 (Shenque), ST25 (Tianshu), and CV12 (Zhongwan) and maintained at 42–45°C using a thermostatically controlled heating pad (Yamato Scientific Co., Ltd., Tokyo, Japan).

#### 2.3.3 BS group

Preheated medical bianstone was used for gentle and continuous warm ironing along the Governor Vessel. The bianstone was uniformly preheated to a stable surface temperature of 42–45°C, consistent with the thermal interface temperature of herbal hot compress. All BS manipulations were performed with a standardized mild physical pressure of 15–20 g, which is a validated non-invasive stimulation intensity for rodent cutaneous thermal intervention without causing mechanical tissue compression or stress damage. Each single longitudinal stroking along the Governor Vessel (from GV14 to GV1) lasted 2 seconds, with a continuous repetitive stroking frequency of 30 strokes per minute throughout the entire intervention period. The total single-session BS intervention duration was strictly maintained at 30 minutes. During the whole BS procedure, rats were gently and fixedly restrained in the customized soft elastic plastic pouch without anesthesia, allowing limited free movement of the head and limbs to minimize stress response, and no obvious struggling, dyspnea, or skin damage was observed. The standardized stroking route, constant temperature, fixed frequency and mild pressure were strictly implemented in every BS intervention to ensure consistent and reproducible stimulation intensity across all experimental animals.

#### 2.3.4 HC + BS group

Received sequential combined intervention: 30 min abdominal herbal hot compress followed immediately by 30 min Governor Vessel bianstone warm ironing, with a total daily intervention duration of 60 min. Bianstone warm ironing in this group used exactly the same standardized parameters (temperature, pressure, stroke duration/frequency, route, restraint) as the BS monotherapy group to ensure operational consistency.

#### 2.3.5 Antagonist + HC + BS group

Received intraperitoneal injection of PKA selective antagonist H89 (10 mg/kg, Sigma-Aldrich, St. Louis, MO, USA, Cat#B1427) 30 min before the 60-minute sequential combined HC + BS treatment, to inhibit the cAMP/PKA signaling pathway. This dose of H-89 (10 mg/kg, i.p.) has been widely used and validated in rodent studies to effectively inhibit central PKA activity and abolish cAMP/PKA-dependent neural function and behavioral modulation without causing overt motor impairment (35, 36), consistent with the *in vivo* PKA inhibition dosage documented in established pharmacological methodologies (37, 38).

### 2.4 Behavioral tests

All behavioral tests were performed in a sound-insulated and dimly lit laboratory 24 h after the last intervention, with 30 min environmental adaptation before testing. All operations were conducted by a trained investigator blinded to group assignments. Behavioral tracking and analysis were performed using ANY-maze software (Stoelting Co., Wood Dale, IL, USA).

#### 2.4.1 Elevated plus maze (EPM)

The EPM apparatus (Stoelting Co., Wood Dale, IL, USA) consists of two open arms (50 cm × 10 cm) and two closed arms (50 cm × 10 cm × 40 cm), elevated 50 cm above the ground. Each rat was placed on the central platform facing an open arm, and the open-arm residence time percentage and open-arm entry percentage within 5 min were recorded and analyzed.

#### 2.4.2 Open field test (OFT)

The open field was a 100 cm × 100 cm × 40 cm black square arena (Stoelting Co., Wood Dale, IL, USA) divided into central and peripheral zones. Each rat was placed in the central zone, and the central zone residence time percentage and total moving distance within 5 min were recorded to evaluate anxiety-like behaviors and spontaneous activity.

#### 2.4.3 Novel object recognition (NOR)

The test was conducted in the same open-field arena (Stoelting Co., Wood Dale, IL, USA) and included three phases: 10 min adaptation phase (free exploration in an empty arena), 10 min familiarization phase (exposure to two identical cubic objects), and 5 min test phase (replacement of one familiar object with a novel spherical object). Object positions were strictly counterbalanced across individual rats to eliminate innate spatial side preference. All test objects were fabricated from non-scented, non-reflective, and non-attractive inert plastic materials to avoid olfactory or textural bias. The recognition index (RI) was calculated as: novel object exploration time/(novel object exploration time + familiar object exploration time).

### 2.5 *In vivo* hippocampal LTP recording

Rats were anesthetized with 3% pentobarbital sodium and fixed on a stereotaxic apparatus (Stoelting Co., Wood Dale, IL, USA). Body temperature was maintained at 37 ± 0.5 ℃ using a heating pad (Yamato Scientific Co., Ltd., Tokyo, Japan). A stimulating electrode was implanted into the Schaffer collateral pathway and a recording electrode was placed in the hippocampal CA1 pyramidal cell layer for field excitatory postsynaptic potential (fEPSP) acquisition. As previously described (39, 40), three sequential electrophysiological detection protocols were performed in order for each rat: input/output (I/O) curve recordings for the evaluation of basal synaptic transmission, paired-pulse ratio (PPR) recordings for the assessment of presynaptic short-term synaptic plasticity, and theta burst stimulation (TBS)-induced LTP recording for the detection of long-term synaptic plasticity. After 20 min stable baseline recording of fEPSP, TBS was delivered to induce LTP. The stimulation intensity was set to elicit 40%–50% of the maximum fEPSP amplitude. The relative increase in fEPSP slope within 60 min after TBS was quantified to assess synaptic plasticity. All electrophysiological signals were collected and analyzed using pClamp 10.7 software (Molecular Devices, San Jose, CA, USA).

### 2.6 ELISA analysis

After behavioral tests, rats were deeply anesthetized, and abdominal aortic blood was collected. Serum was separated by centrifugation at 3000 r/min for 15 min using a centrifuge (Eppendorf Centrifuge 5810R, Eppendorf AG, Hamburg, Germany). Hippocampal tissues were rapidly dissected and homogenized in pre-cooled PBS, followed by centrifugation at 12000 r/min for 10 min to collect supernatant. All serum and tissue supernatant indexes were strictly quantified using commercial ELISA kits according to the manufacturer's instructions.

Serum detected indexes included intestinal oxidative stress markers: MDA and SOD; intestinal barrier markers: DAO, D-lactic acid and LPS; inflammatory cytokines interleukin-1β (IL-1β), tumor necrosis factor-α (TNF-α); HPA axis hormones corticotropin-releasing hormone (CRH), adrenocorticotropic hormone (ACTH), corticosterone (CORT). Hippocampal detected indexes included cAMP, 5-hydroxytryptamine (5-HT), DA, Glu, and GABA, with the Glu/GABA ratio calculated for each sample.

Serum IL-1β (Cat#CSB-E08055r), TNF-α (Cat#CSB-E11987r), CRH (Cat#CSB-E08038r), ACTH (Cat#CSB-E06875r), CORT (Cat#CSB-E07014r), MDA (Cat# CSB-E13354r), SOD (Cat#CSB-E13355r), DAO (Cat#CSB-E14393r), D-lactic acid (Cat#CSB-E14394r) and LPS (Cat#CSB-E14402r) were measured with kits from CUSABIO (Wuhan, China). Hippocampal cAMP (Cat#H106-1), 5-hydroxytryptamine (5-HT; Cat#H104-1-2), DA (Cat#H091-1-2), Glutamate (Cat#A074-1-2), and GABA (Cat#A035-1-2) were measured with kits from Nanjing Jiancheng Bioengineering Institute (Nanjing, China). All tissue supernatant protein concentrations were normalized using the BCA protein assay kit (Beyotime Biotechnology, Shanghai, China) to eliminate differences in sample loading. All biochemical indicators were finally calibrated by total protein content.

### 2.7 Statistical analysis

All data were expressed as mean ± standard deviation (mean ± SD). Statistical analysis was performed using SPSS 26.0 software (IBM Corp., Armonk, NY, USA). Data normality and homogeneity of variance were verified by Shapiro–Wilk and Levene's tests prior to analysis of variance (ANOVA). One-way ANOVA was used for multi-group comparison, and Tukey's *post hoc* test was used for pairwise multiple comparison. *P* < 0.05 was considered statistically significant, and *P* < 0.01 was considered highly statistically significant.

## 3 Results

### 3.1 Serum intestinal oxidative stress and intestinal barrier index levels

Successful ML model establishment was confirmed by significant increases in MLN wet weight and the normalized MLN index. One-way ANOVA revealed highly significant intergroup differences in MLN weight (*F*_(5,42)_ = 52.67, *P* < 0.001) and MLN index (*F*_(5,42)_ = 48.92, *P* < 0.001). Compared with the Control group, Model group rats exhibited significantly increased MLN weight (222.0 ± 13.5 mg vs. 120.0 ± 8.2 mg; *P* < 0.01) and MLN index (0.70 ± 0.05% vs. 0.38 ± 0.02%; *P* < 0.01), indicating marked mesenteric lymph node hyperplasia and successful establishment of the ML model. Notably, single BS intervention showed no significant ameliorative effects on MLN weight (212.5 ± 13.9 mg) and MLN index (0.68 ± 0.05%) (*P* > 0.05 vs. Model). HC treatment significantly reduced MLN weight to 188.3 ± 10.6 mg and MLN index to 0.56 ± 0.03% (*P* < 0.05 vs. Model). Notably, HC + BS combined treatment almost completely normalized MLN weight (133.8 ± 9.7 mg) and MLN index (0.40 ± 0.03%), returning to near-Control levels (*P* < 0.01 vs. Model), with efficacy significantly superior to both HC and BS monotherapy (both *P* < 0.05). Administration of H89 markedly abrogated the therapeutic effect of HC + BS, leading to MLN weight (189.4 ± 14.8 mg) and MLN index (0.59 ± 0.05%) that were significantly higher than those in the HC + BS group (*P* < 0.05 vs. HC + BS).

**Figure 1:**
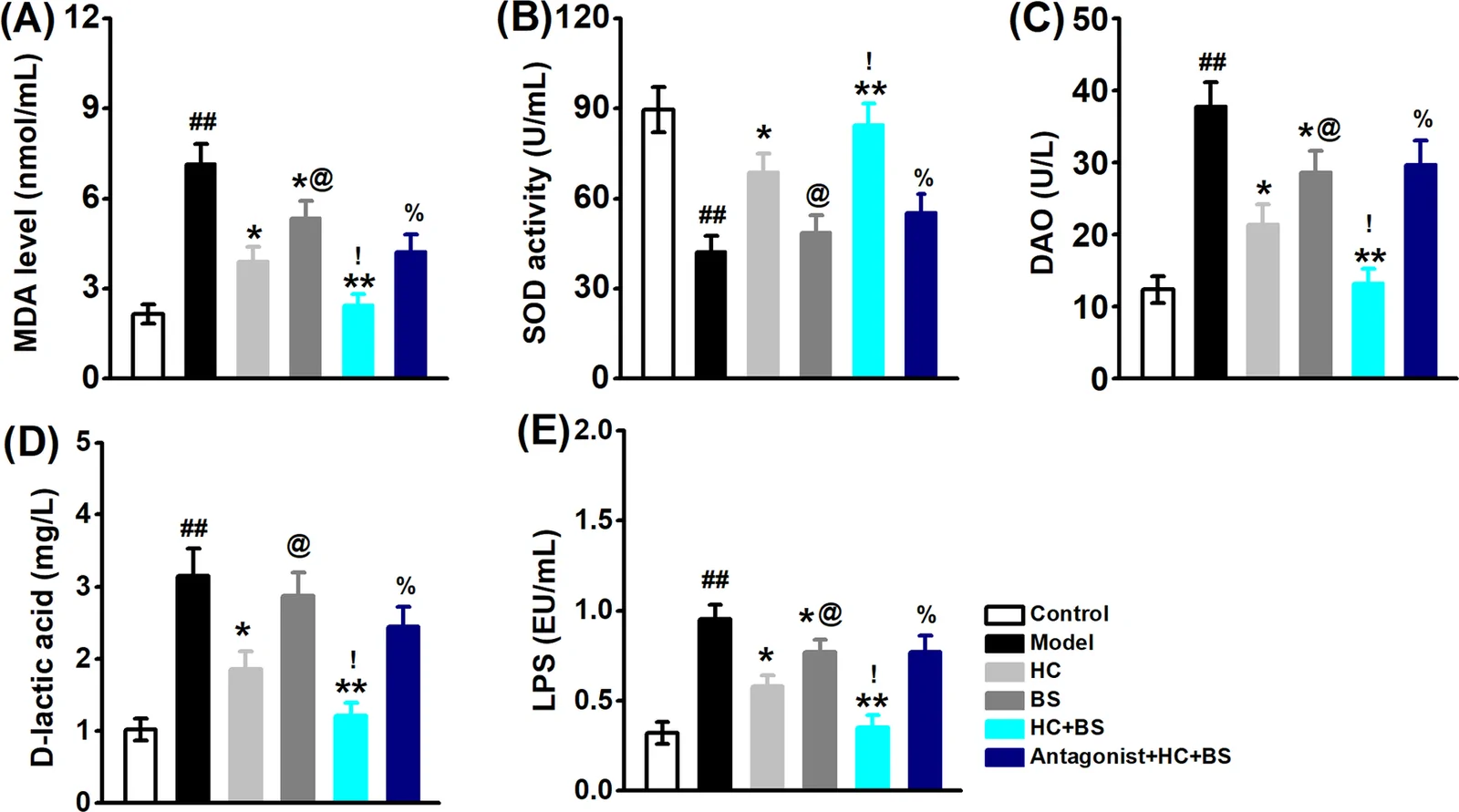
Effects of HC, BS and their combined intervention on serum intestinal oxidative stress and intestinal barrier biomarkers in young rats with ML established during puberty. **(A)** Serum malondialdehyde (MDA) concentration. **(B)** Serum superoxide dismutase (SOD) activity. **(C)** Serum diamine oxidase (DAO) level. **(D)** Serum D-lactic acid concentration. **(E)** Serum lipopolysaccharide (LPS) level. All data are presented as mean ± SD, *n* = 8 per group. Statistical comparisons were performed by one-way ANOVA followed by Tukey's *post hoc* test. ##*P* < 0.01 vs. Control group; **P < 0.01, *P < 0.05 vs. Model group; @*P* < 0.05 vs. HC group; !*P* < 0.05 vs. HC and BS monotherapy groups; %*P* < 0.05 vs HC + BS combination group.

The indices of intestinal oxidative stress (MDA, SOD) and intestinal barrier integrity (DAO, D-lactic acid, LPS) in serum are summarized in Figure 1, and the intergroup differences were analyzed by one-way ANOVA. One-way ANOVA revealed highly significant intergroup differences in serum oxidative stress indexes MDA (*F*_(5,42)_ = 47.23, *P* < 0.001), SOD (*F*_(5,42)_ = 43.58, *P* < 0.001) and intestinal barrier indexes DAO (*F*_(5,42)_ = 41.96, *P* < 0.001), D-lactic acid (*F*_(5,42)_ = 38.75, *P* < 0.001), LPS (*F*_(5,42)_ = 45.12, *P* < 0.001). Compared with the Control group, Model group rats exhibited severe intestinal oxidative stress injury and intestinal barrier dysfunction, manifested as significantly increased serum MDA, DAO, D-lactic acid and LPS levels, and markedly decreased SOD activity (all *P* < 0.01), indicating successful induction of intestinal redox imbalance and mucosal barrier damage in ML model rats.

BS treatment significantly reduced serum MDA, DAO and LPS levels and slightly elevated SOD activity (*P* < 0.05 vs. Model), but showed no significant improvement on D-lactic acid (*P* > 0.05 vs. Model), and its regulatory effects on all oxidative stress and intestinal barrier indexes were significantly weaker than HC (*P* < 0.05 *vs.* HC). HC treatment exerted prominent protective effects on intestinal redox balance and barrier function, which significantly decreased MDA, DAO, D-lactic acid, LPS levels and restored SOD activity (all *P* < 0.05 *vs.* Model).

Notably, HC + BS combined treatment completely reversed intestinal oxidative stress and barrier dysfunction, making all indexes close to normal control levels (*P* < 0.01 *vs.* Model), and the efficacy was significantly superior to both HC and BS monotherapy (*P* < 0.05). Intervention with PKA antagonist H89 fully abrogated the protective effects of HC + BS on all detected intestinal oxidative stress and barrier biomarkers. Compared to the HC + BS group, the Antagonist + HC + BS group exhibited markedly elevated serum MDA, DAO, D-lactic acid and LPS concentrations, alongside suppressed SOD activity, demonstrating severe recurrence of intestinal lipid peroxidation injury and intestinal hyperpermeability (all *P* < 0.05 *vs.* HC + BS).

### 3.2 Serum inflammatory cytokine levels

**Figure 2:**
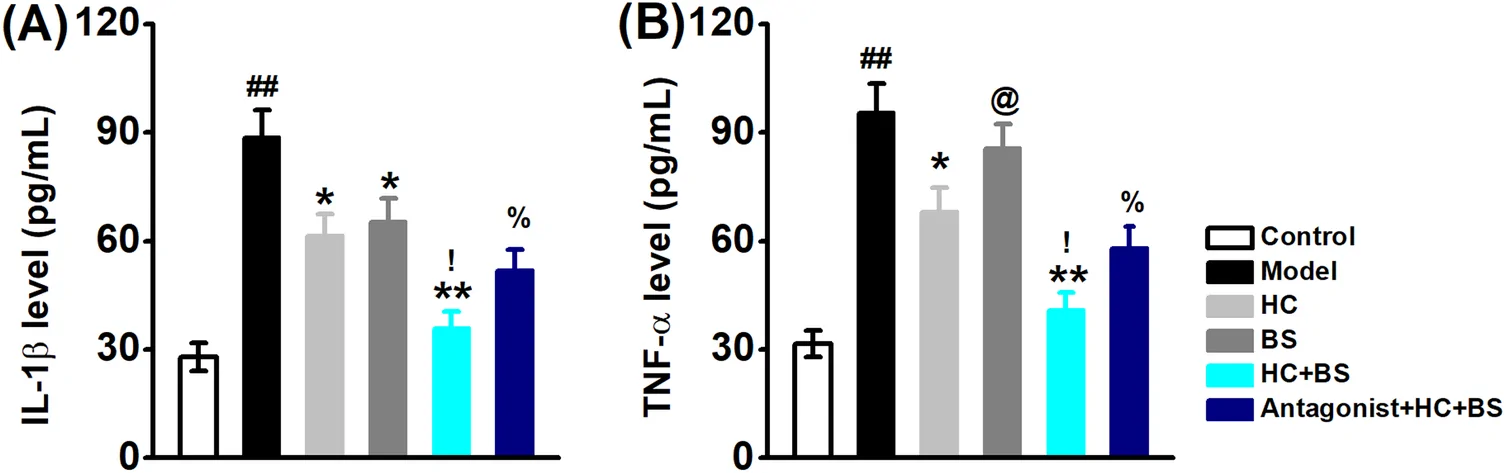
Effects of HC, BS and their combined intervention on serum pro-inflammatory cytokines in young rats with ML established during puberty. **(A)** Serum interleukin-1β (IL-1β) level. **(B)** Serum tumor necrosis factor-α (TNF-α) level. All data are presented as mean ± SD, *n* = 8 per group. Statistical comparisons were performed by one-way ANOVA followed by Tukey's *post hoc* test. ##*P* < 0.01 vs. Control group; **P < 0.01, *P < 0.05 vs. Model group; @*P* < 0.05 vs. HC group; !*P* < 0.05 vs. HC and BS monotherapy groups; %*P* < 0.05 vs. HC + BS combination group.

Serum levels of pro-inflammatory cytokines IL-1β and TNF-α are presented in Figure 2, with statistical analysis shown below. One-way ANOVA revealed highly significant intergroup differences in serum IL-1β (*F*_(5,42)_ = 42.76, *P* < 0.001) and TNF-α (*F*_(5,42)_ = 39.14, *P* < 0.001). Compared with the Control group, serum IL-1β and TNF-α levels were markedly increased in the Model group (*P* < 0.01), indicating severe systemic inflammation induced by intestinal oxidative stress and barrier damage. BS treatment significantly reduced IL-1β and TNF-α levels relative to Model (*P* < 0.05), but its effect on IL-1β was significantly weaker than HC (*P* < 0.05 *vs.* HC). HC treatment induced pronounced reductions in both inflammatory cytokines (*P* < 0.05 *vs.* Model). HC + BS combined treatment normalized IL-1β and TNF-α to near-control levels (*P* < 0.01 *vs.* Model), with efficacy significantly better than both HC and BS alone (*P* < 0.05). Pre-administration of PKA antagonist H89 markedly blunted the anti-inflammatory capacity of HC + BS, resulting in robust upregulation of serum pro-inflammatory IL-1β and TNF-α compared with the HC + BS group (*P* < 0.05 *vs.* HC + BS), confirming blockade of the combined therapy's peripheral anti-inflammatory action.

### 3.3 Serum HPA axis hormone levels

**Figure 3:**
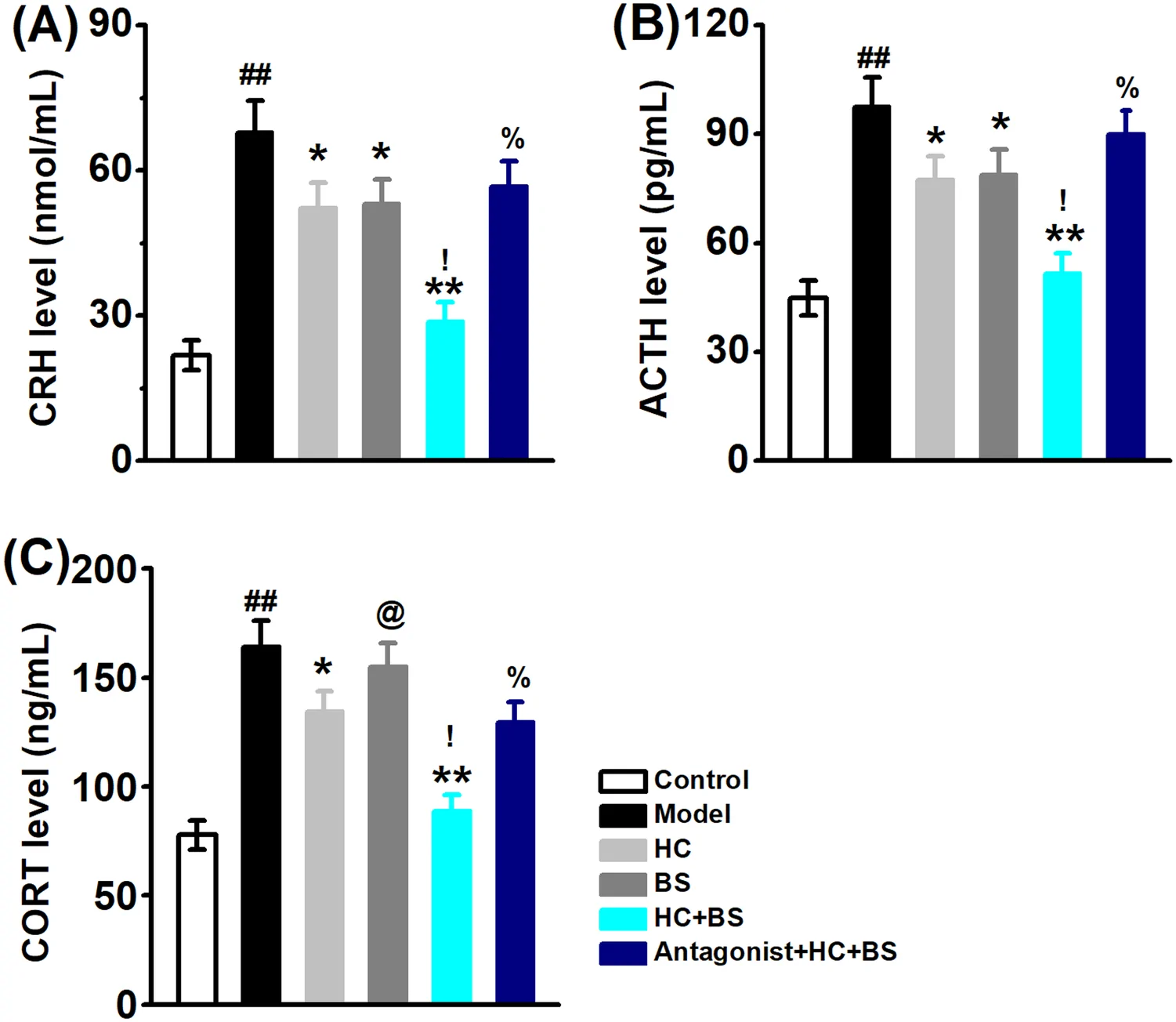
Effects of HC, BS and their combined intervention on serum HPA axis hormone levels in young rats with ML established during puberty. **(A)** Serum corticotropin-releasing hormone (CRH) concentration. **(B)** Serum adrenocorticotropic hormone (ACTH) concentration. **(C)** Serum corticosterone (CORT) concentration. All data are presented as mean ± SD, *n* = 8 per group. Statistical comparisons were performed by one-way ANOVA followed by Tukey's *post hoc* test. ##*P* < 0.01 vs. Control group; **P < 0.01, *P < 0.05 vs. Model group; @*P* < 0.05 vs. HC group; !*P* < 0.05 vs. HC and BS monotherapy groups; %*P* < 0.05 vs. HC + BS combination group.

Serum HPA axis hormones CRH, ACTH and CORT are shown in Figure 3. Highly significant intergroup differences were detected in CRH (*F*_(5,42)_ = 45.82, *P* < 0.001), ACTH (*F*_(5,42)_ = 41.36, *P* < 0.001), and CORT (*F*_(5,42)_ = 49.71, *P* < 0.001). The Model group exhibited marked HPA axis hyperactivation with elevated CRH, ACTH, and CORT (*P* < 0.01 *vs.* Control). BS significantly lowered CRH and ACTH (*P* < 0.05 *vs.* Model), but was significantly less effective than HC in reducing CORT (*P* < 0.05 *vs.* HC). HC consistently suppressed excessive HPA axis hormone secretion (*P* < 0.05 *vs.* Model). HC + BS treatment restored CRH, ACTH, and CORT close to normal levels (*P* < 0.01 *vs.* Model), with efficacy significantly superior to both monotherapies (*P* < 0.05). PKA antagonist H89 completely reversed the inhibitory effect of HC + BS on overactive HPA axis endocrine secretion; the Antagonist + HC + BS group displayed significantly higher circulating CRH, ACTH and CORT concentrations than the HC + BS group (*P* < 0.05 *vs.* HC + BS), eliminating the combined intervention's capacity to normalize stress hormone homeostasis.

### 3.4 Anxiety-like behaviors, cognitive function and hippocampal LTP

**Figure 4:**
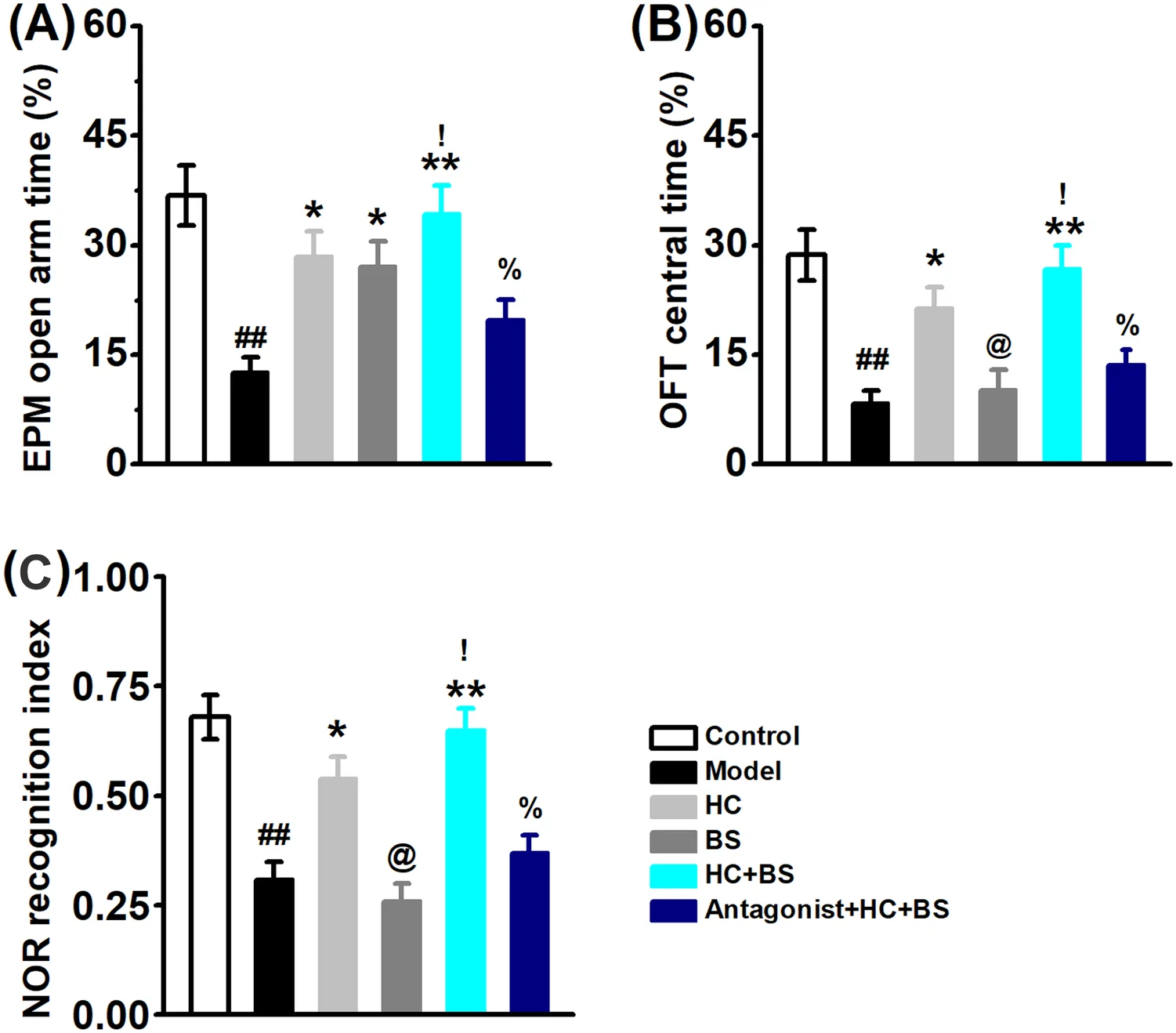
Effects of HC, BS and their combined intervention on anxiety-like behaviors and cognitive performance in young rats with ML established during puberty. **(A)** Percentage of open-arm residence time in the elevated plus maze (EPM) test. **(B)** Percentage of central zone residence time in the open field test (OFT). **(C)** Recognition index calculated from the novel object recognition (NOR) test. All data are presented as mean ± SD, *n* = 8 per group. Statistical comparisons were performed by one-way ANOVA followed by Tukey's *post hoc* test. ##*P* < 0.01 vs. Control group; **P < 0.01, *P < 0.05 vs. Model group; @*P* < 0.05 vs. HC group; !*P* < 0.05 vs. HC and BS monotherapy groups; %*P* < 0.05 vs. HC + BS combination group.

**Figure 5:**
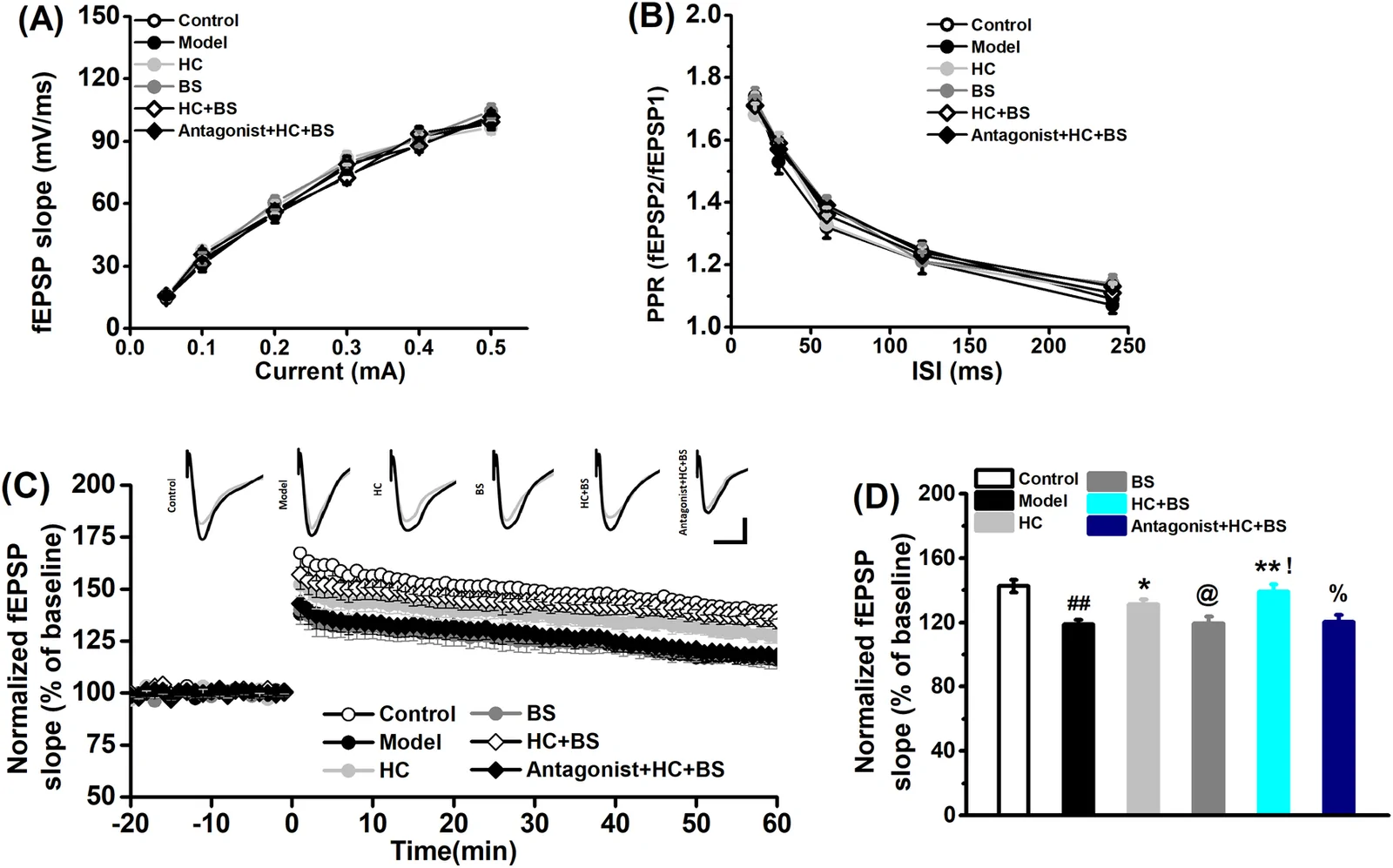
Effects of HC, BS, and their combination on hippocampal basal synaptic transmission, presynaptic short-term plasticity and LTP in young rats with ML established during puberty. **(A)** I/O curves of fEPSP slope against stimulation current. **(B)** PPR curves across different inter-stimulus intervals (ISI). **(C)** Time course of normalized fEPSP slope over 60 min after theta burst stimulation (TBS) in each group (mean ± SD, *n* = 8). Insets show representative superimposed fEPSP traces. The gray trace indicates a baseline fEPSP recorded before TBS, while the black trace represents a steady-state fEPSP obtained during the final 20 min of post-TBS recording. Vertical scale bar: 0.2 mV; horizontal scale bar: 5 ms. **(D)** Comparison of the average normalized fEPSP slope during the last 20 min of LTP recording in each group (mean ± SD, *n* = 8). Statistical comparisons were performed by one-way ANOVA followed by Tukey's *post hoc* test. ##*P* < 0.01 vs. Control group; **P < 0.01, *P < 0.05 vs. Model group; @*P* < 0.05 vs. HC group; !*P* < 0.05 vs. HC and BS monotherapy groups; %*P* < 0.05 vs. HC + BS combination group.

Behavioral performance in EPM, OFT and NOR tests is presented in Figure 4, while *in vivo* hippocampal synaptic plasticity are illustrated in Figure 5. One-way ANOVA showed highly significant intergroup differences in EPM open-arm time percentage (*F*_(5,42)_ = 38.42, *P* < 0.001), OFT central zone time percentage (*F*_(5,42)_ = 35.76, *P* < 0.001), NOR recognition index (*F*_(5,42)_ = 40.19, *P* < 0.001), and hippocampal LTP relative fEPSP slope increase (Figure 5D, *F*_(5,42)_ = 44.33, *P* < 0.001). However, no significant main effect of group was observed in either I/O curve fEPSP slope (*F*_(5,42)_ = 1.12, *P* > 0.05) or PPR (*F*_(5,42)_ = 0.98, *P* > 0.05), indicating comparable basal synaptic transmission and presynaptic short-term plasticity across all six experimental groups. Compared with Control, Model rats displayed severe anxiety-like behaviors, impaired recognition memory, and significant suppression of hippocampal LTP synaptic plasticity (all *P* < 0.01). BS only significantly improved EPM open-arm time (*P* < 0.05 *vs.* Model), but showed no obvious improvement in OFT central time, NOR recognition index and LTP, and was significantly weaker than HC in these indicators (*P* < 0.05 *vs.* HC). HC significantly improved all behavioral and electrophysiological indexes (*P* < 0.05 *vs.* Model). HC + BS treatment nearly fully restored emotional-cognitive function and synaptic plasticity (*P* < 0.01 *vs.* Model), with markedly stronger effects than both HC and BS alone (*P* < 0.05). Blockade of PKA signaling via H89 fully abolished all behavioral and synaptic protective phenotypes induced by HC + BS: relative to HC + BS rats, Antagonist + HC + BS rats exhibited sharply reduced EPM open-arm residence ratio, shortened OFT central zone exploration time, declined NOR recognition index, and prominent attenuation of hippocampal LTP fEPSP potentiation (all *P* < 0.05 *vs.* HC + BS), returning anxiety, memory deficits and synaptic dysfunction to levels similar to untreated model animals.

### 3.5 Hippocampal cAMP and neurotransmitter levels

**Figure 6:**
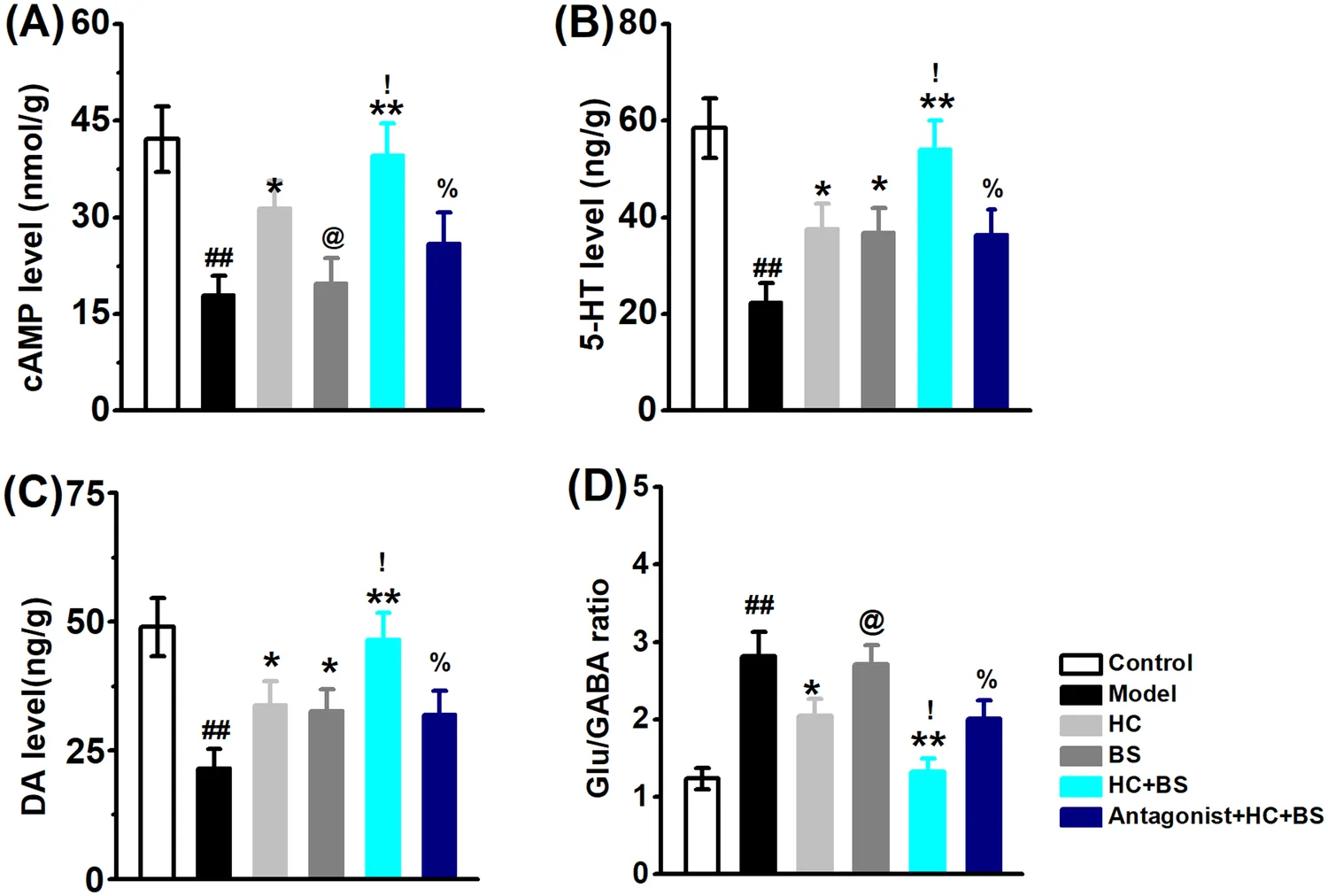
Effects of HC, BS and their combined intervention on hippocampal cAMP, monoamine neurotransmitters and excitatory-inhibitory balance in young rats with ML established during puberty. **(A)** Hippocampal cyclic adenosine monophosphate (cAMP) level. **(B)** Hippocampal 5-hydroxytryptamine (5-HT) level. **(C)** Hippocampal dopamine (DA) level. **(D)** Glutamate/GABA (Glu/GABA) ratio in hippocampal tissue. All data are presented as mean ± SD, *n* = 8 per group. Statistical comparisons were performed by one-way ANOVA followed by Tukey's *post hoc* test. ##*P* < 0.01 vs. Control group; **P < 0.01, *P < 0.05 vs. Model group; @*P* < 0.05 vs. HC group; !*P* < 0.05 vs. HC and BS monotherapy groups; %*P* < 0.05 vs. HC + BS combination group.

Hippocampal cAMP, monoamine neurotransmitters (5-HT, DA), Glu and GABA, as well as the calculated Glu/GABA ratio, are summarized in Figure 6. One-way ANOVA revealed highly significant intergroup differences in hippocampal cAMP (*F*_(5,42)_ = 46.85, *P* < 0.001), 5-HT (*F*_(5,42)_ = 40.28, *P* < 0.001), DA (*F*_(5,42)_ = 39.62, *P* < 0.001) and Glu/GABA ratio (*F*(_5,42)_ = 44.19, *P* < 0.001). Compared with the Control group, the Model group exhibited markedly decreased hippocampal cAMP, 5-HT, DA and increased Glu/GABA ratio (*P* < 0.01). BS significantly elevated 5-HT and DA (*P* < 0.05 *vs.* Model), but was significantly less effective than HC in increasing cAMP and reducing Glu/GABA ratio (*P* < 0.05 *vs.* HC). HC robustly restored hippocampal cAMP, monoamine neurotransmitters, and excitatory-inhibitory balance (*P* < 0.05 *vs.* Model). HC + BS treatment normalized all hippocampal neurochemical indexes (*P* < 0.01 *vs.* Model), with efficacy significantly superior to both monotherapies (*P* < 0.05). PKA antagonist H89 reversed the regulatory effects of HC + BS on every measured hippocampal neurochemical marker: the Antagonist + HC + BS group showed significantly lower hippocampal cAMP, 5-HT and DA content, alongside a markedly elevated Glu/GABA excitatory-inhibitory ratio compared with the HC + BS group (all *P* < 0.05 *vs.* HC + BS), disrupting the balanced neurotransmitter environment recovered by combined herbal and bianstone therapy.

## 4 Discussion

The present study successfully established a rat model of ML via repeated LPS injection, which was quantitatively validated by significantly increased macroscopic MLN wet weight and normalized MLN organ index, the macroscopic indicators for evaluating mesenteric lymph node inflammatory hyperplasia in rodent ML models. This modeling approach has been well documented to induce persistent intestinal inflammation, mesenteric lymph node enlargement, and lymphatic tissue dysfunction in rodents (28–32). Pediatric ML triggers persistent intestinal inflammation, which can disrupt the bidirectional gut-brain axis and induce excessive HPA axis activation (3, 20). Intestinal oxidative stress imbalance is a key initiating factor of ML-induced intestinal injury: excessive MDA accumulation aggravates intestinal epithelial cell lipid peroxidation and apoptosis, while decreased SOD antioxidant activity weakens the intestinal mucosal antioxidant defense system, further exacerbating inflammatory infiltration. Damaged intestinal barrier increases mucosal permeability, leading to massive release of intestinal LPS into blood circulation, accompanied by elevated serum DAO and D-lactic acid levels, which are specific biomarkers of intestinal mucosal epithelial injury (5, 41). The persistent peripheral oxidative stress damage, barrier dysfunction and inflammatory activation further impair hippocampal cAMP signaling, reduce monoamine neurotransmitter production, disrupt glutamatergic/GABAergic balance, and compromise synaptic plasticity, ultimately promoting anxiety-like behaviors and cognitive dysfunction (42–44). The present study demonstrated that HC, BS, and their combination effectively alleviated systemic inflammation, normalized HPA axis hormones, restored hippocampal cAMP and neurotransmitter homeostasis, enhanced synaptic plasticity, and improved anxiety and recognition memory in pubertal rats subjected to LPS-induced mesenteric inflammation.

The herbal formula used for hot compress contains multiple active ingredients with antioxidant and intestinal barrier protective effects. Rhizoma Atractylodis Preparata can restore intestinal barrier function and suppress inflammatory signaling (45); Rhizoma Atractylodis Macrocephalae Preparata exerts anti-oxidative and gastroprotective activities (46); Pericarpium Citri Reticulatae is rich in hesperidin, a typical flavonoid compound that inhibits NLRP3 inflammasome activation to alleviate intestinal inflammation (47); Fructus Crataegi Carbonisatus contains chlorogenic acid and hawthorn flavonoids that effectively scavenge intestinal reactive oxygen species, reduce MDA generation, and downregulate pro-inflammatory cytokines including IL-1β and TNF-α (48); Fructus Hordei Germinatus Preparatus possesses malt oligosaccharides that modulate gut microbiota composition and promote short-chain fatty acid production to maintain intestinal microecological homeostasis (49); Endothelium Corneum Gigeriae Galli Preparatum improves gastrointestinal motility by promoting gastric emptying and intestinal peristalsis (50); Semen Coicis Crudum contains coixenolide with prominent anti-inflammatory and immunomodulatory effects, cooperating with hawthorn flavonoids to enhance intestinal antioxidant capacity (51); Rhizoma Dioscoreae Preparata is abundant in diosgenin, which protects intestinal epithelium against oxidative injury and tight junction disruption (52); Rhizoma Curcumae Vinegar-processed contains curcuminoids, serving as potent inhibitors of cyclooxygenase 2 (COX-2) and inducible nitric oxide synthase (iNOS) to block inflammatory cascade activation (53); Fructus Toosendan Preparatum contains toosendanin, which suppresses mast cell degranulation and reduces visceral hypersensitivity to relieve abdominal discomfort (54); and Spica Prunellae is enriched with rosmarinic acid and ursolic acid, which inhibit NF-κB and MAPK signaling pathways, alleviate mesenteric lymph node hyperplasia, and exert reliable anti-inflammatory and immunomodulatory effects on abdominal lymphatic disorders (55). This multi-component formula provides a solid phytochemical basis for HC to improve intestinal redox balance, repair intestinal mucosal barrier, block LPS leakage and peripheral inflammatory cascade, regulate intestinal microecology and visceral sensitivity, and thereby improving gut-brain axis dysfunction (56–60).

A prominent efficacy difference was observed between HC and BS monotherapies in regulating intestinal oxidative stress and barrier function. BS alone only produced limited improvements in partial oxidative stress and intestinal barrier indexes, with no significant regulatory effect on D-lactic acid, and its efficacy in reducing intestinal oxidative damage and improving barrier permeability was significantly weaker than HC. This indicates that BS only plays an auxiliary regulatory role in systemic circulation and central mood pathways, but cannot fundamentally reverse intestinal peripheral oxidative stress injury and mucosal barrier damage caused by ML, which is the core pathological link of gut-brain axis dysfunction. This is consistent with previous reports that non-pharmacological warm stimulation can partially modulate autonomic activity but has limited effect on intestinal mucosal oxidative injury and barrier repair (61, 62). In contrast, HC alone produced broad and consistent improvements across all measured parameters, which is consistent with its localized action on abdominal acupoints and inflamed mesenteric tissue to suppress inflammatory signaling at the source and aligns with clinical observations that herbal warm compress at CV8 effectively alleviates mesenteric inflammation and associated abdominal pain (63, 64).

More importantly, HC + BS combined treatment yielded significant synergistic effects, achieving nearly complete normalization of intestinal oxidative stress injury, intestinal barrier dysfunction, systemic inflammation, HPA axis hyperactivity, neurochemical disturbance, synaptic plasticity damage and behavioral abnormalities, with efficacy significantly superior to both monotherapies. The synergistic mechanism lies in the complementary advantages of the two therapies: HC exerts peripheral protective effects by improving intestinal redox balance, repairing intestinal barrier and blocking peripheral pathological stimulation, while BS assists in regulating central neuroendocrine and gut-brain axis circulation, jointly reversing the whole-process dysfunction of the gut-brain-HPA axis (65–67). Mechanistically, the beneficial effects of combined treatment on intestinal oxidative stress, intestinal barrier and gut-brain axis homeostasis were completely blocked by PKA antagonist H89, indicating that the cAMP/PKA signaling pathway is an important molecular basis for combined therapy to improve intestinal peripheral injury and central emotional-cognitive dysfunction (68, 69). Notably, the cAMP/PKA pathway exhibits distinct compartment-specific functional characteristics in peripheral intestinal and central hippocampal tissues (18, 70–72), which explains the dual gut-brain regulatory effects of HC + BS treatment. In the peripheral intestinal compartment, moderately activated cAMP/PKA signaling serves as a key protective cascade to suppress intestinal epithelial lipid peroxidation, inhibit local inflammatory infiltration, and maintain intestinal mucosal barrier integrity, thereby eliminating the peripheral pathological source of gut-brain axis dysfunction (37, 70, 73–75). In this study, this peripheral cAMP/PKA-dependent protective effect is mainly driven by localized herbal hot compress intervention. In the central hippocampal compartment, cAMP/PKA activation modulates the homeostasis of monoamine neurotransmitters and glutamatergic-GABAergic balance, and facilitates hippocampal synaptic LTP formation, which directly rescues inflammation-induced anxiety-like behaviors and cognitive impairment (76–80), and this central neuromodulatory effect is synergistically enhanced by bianstone warm ironing. Consistent with previous authoritative studies on gut-brain cAMP/PKA signaling, the peripheral and central cAMP/PKA cascades mediate relatively independent but mutually coordinated therapeutic functions: peripheral cAMP/PKA activation targets intestinal pathological damage, while central cAMP/PKA activation targets neural and behavioral dysfunction, and both are indispensable for the holistic gut-brain regulatory efficacy of HC + BS combined therapy. The physiological elevation of cAMP/PKA induced by HC + BS was moderate and beneficial for intestinal barrier repair and synaptic homeostasis. However, pharmacological blockage of PKA by H89 disrupted the optimal cAMP/PKA signaling amplitude, induced negative feedback regulation, reversed the therapeutic effects, and ultimately abrogated the gut-brain-HPA axis protective effects of combined intervention.

Notably, no significant intergroup differences were detected in I/O curves reflecting basal synaptic transmission or PPR representing presynaptic short-term plasticity among all six groups. Peripheral intestinal/mesenteric inflammation has been shown to selectively impair hippocampal late-phase LTP while sparing basal synaptic transmission and presynaptic release properties. For instance, TNBS-induced colonic inflammation did not alter paired-pulse ratios or mEPSC frequencies, indicating intact presynaptic function, yet significantly attenuated hippocampal LTP, with basal fEPSP slopes largely preserved (81). Similarly, acute pro-inflammatory challenges preferentially disrupt protein-synthesis–dependent late-phase LTP via NLRP3/25-HC signaling without compromising basic transmission (82), a pattern consistent with the observation that IL-1β modulates plasticity over basal release (83). In the present study, pubertal ML mirrored these selective effects, primarily compromising hippocampal late-phase synaptic plasticity while leaving fundamental transmission and presynaptic parameters intact. Likewise, herbal hot compress combined with bianstone warm ironing exerted its protective effects specifically by restoring TBS-induced LTP, rather than modifying basal excitatory transmission or presynaptic short-term plasticity. The unaltered I/O and PPR profiles exclude non-specific changes in basal synaptic function and further verify that the observed improvement in cognitive function is attributable to the restoration of activity-dependent synaptic plasticity.

Nevertheless, several limitations should be acknowledged. First, although the present study detected hippocampal cAMP levels and applied H89-mediated PKA pharmacological inhibition to verify pathway function, intervention with a single pharmacological antagonist is insufficient to fully confirm cAMP/PKA as the primary therapeutic mechanism. Direct assessment of PKA kinase activity and downstream signaling molecules is required to provide more rigorous evidence for pathway activation and specificity. Therefore, the mechanistic conclusions of the current study are interpreted with appropriate caution, and the potential involvement of other cAMP-dependent signaling cascades cannot be excluded, which will be further clarified in future investigations. Second, this study did not directly assess intestinal mucosal barrier function (including tight junction proteins such as occludin and claudins) or intestinal permeability, nor did it characterize gut microbiota composition. Given the central role of the gut barrier and microbiota in gut-brain axis signaling (3), future studies will further verify the protective mechanism of combined therapy on intestinal mucosal structure and oxidative stress at the tissue and molecular levels. Third, the precise molecular mechanisms underlying the effects of bianstone warm ironing on intestinal oxidative stress remain largely unexplored. Further targeted research on BS is needed. Fourth, the ML model cannot fully replicate the natural pathological progression of clinical pediatric ML. In addition, only male pubertal rats were enrolled in the current experiment to exclude estrous cycle-mediated hormonal interference on intestinal inflammation and HPA axis responses (84, 85). Therefore, the research conclusions of this study are only generalizable to pubertal male rats. Sex differences in therapeutic efficacy and gut-brain-HPA axis regulation require further verification with age-matched female animals in future work, limiting the generalization of findings (86, 87).

In summary, herbal hot compress combined with bianstone warm ironing significantly ameliorates intestinal oxidative stress imbalance, intestinal barrier damage, intestinal inflammation, HPA axis hyperactivity, hippocampal neurochemical disturbance, synaptic dysfunction, and accompanying anxiety and cognitive deficits in pubertal ML rats, with prominent synergistic efficacy superior to monotherapy. Activation of the cAMP/PKA signaling pathway mediates the dual regulatory effects of combined therapy on intestinal peripheral protection and central nerve function improvement. These findings enrich the mechanistic connotation of TCM “treating brain disorders from intestinal lesions” and provide preclinical support for the clinical application of combined external thermal therapy in pediatric gut-derived emotional-cognitive complications.

## 5 Conclusion

In conclusion, the combined application of herbal hot compress and bianstone warm ironing produces synergistic therapeutic effects against ML-induced gut-brain-HPA axis dysfunction when ML is established during puberty and assessed in young rats. This effect is mediated by the cAMP/PKA signaling pathway, as evidenced by complete blockade of the therapeutic effects by the PKA antagonist H-89. These findings provide preclinical evidence supporting the clinical translation of combined external thermal therapy for managing gut-derived emotional-cognitive comorbidities in children, and highlight the cAMP/PKA pathway as a potential therapeutic target for future intervention strategies.

## Data Availability

The datasets presented in this study can be found in online repositories. The names of the repository/repositories and accession number(s) can be found below: https://figshare.com/, https://doi.org/10.6084/m9.figshare.32848829.
